# Zinc Biofortification in Food Crops Could Alleviate the Zinc Malnutrition in Human Health

**DOI:** 10.3390/molecules26123509

**Published:** 2021-06-09

**Authors:** Subhashisa Praharaj, Milan Skalicky, Sagar Maitra, Preetha Bhadra, Tanmoy Shankar, Marian Brestic, Vaclav Hejnak, Pavla Vachova, Akbar Hossain

**Affiliations:** 1 Department of Agronomy, Centurion University of Technology and Management, Pralakhemundi 761211, India; subhashisa.praharaj@cutm.ac.in (S.P.); sagar.maitra@cutm.ac.in (S.M.); tanmoy@cutm.ac.in (T.S.); 2 Department of Botany and Plant Physiology, Faculty of Agrobiology, Food, and Natural Resources, Czech University of Life Sciences Prague, Kamycka 129, 165 00 Prague, Czech Republic; marian.brestic@uniag.sk (M.B.); hejnak@af.czu.cz (V.H.); vachovap@af.czu.cz (P.V.); 3 Department of Biotechnology, Centurion University of Technology and Management, Pralakhemundi 761211, India; preetha.bhadra@cutm.ac.in; 4 Department of Plant Physiology, Slovak University of Agriculture, Nitra, Tr. A. Hlinku 2, 949 01 Nitra, Slovakia; 5 Department of Agronomy, Bangladesh Wheat and Maize Research Institute, Dinajpur 5200, Bangladesh

**Keywords:** agronomic biofortification, genetic biofortification, malnutrition, micronutrient, zinc

## Abstract

Micronutrient malnutrition is a global health issue and needs immediate attention. Over two billion people across the globe suffer from micronutrient malnutrition. The widespread zinc (Zn) deficiency in soils, poor zinc intake by humans in their diet, low bioavailability, and health consequences has led the research community to think of an economic as well as sustainable strategy for the alleviation of zinc deficiency. Strategies like fortification and diet supplements, though effective, are not economical and most people in low-income countries cannot afford them, and they are the most vulnerable to Zn deficiency. In this regard, the biofortification of staple food crops with Zn has been considered a useful strategy. An agronomic biofortification approach that uses crop fertilization with Zn-based fertilizers at the appropriate time to ensure grain Zn enrichment has been found to be cost-effective, easy to practice, and efficient. Genetic biofortification, though time-consuming, is also highly effective. Moreover, a Zn-rich genotype once developed can also be used for many years without any recurring cost. Hence, both agronomic and genetic biofortification can be a very useful tool in alleviating Zn deficiency.

## 1. Introduction

Food and nutritional security are key to human health. Food insecurity, imbalanced diet, consumption of food grains with poor nutritional quality, lack of dietary diversity, etc. negatively affect human health [1,2]. In fact, food and nutritional insecurity may further deteriorate diet quality; thus, increasing the danger of undernutrition as well as obesity [3]. The increase in the cultivation of cereal crops and cash crops in the intensive cropping systems have caused a reduction of diversity in the diet as well as micronutrient uptake [4]. This is especially true in rural areas of developing countries, where the human diet is largely based on cereal. The green revolution era that introduced input responsive and high yielding varieties of some fine cereals like wheat and rice made agriculture profitable for farmers. This led the farmers to grow highly productive and economically rewarding cereal crops, with a simultaneous decrease in the area growing pulses [4]. The low dietary diversity is evident from the fact that, out of 7000 species ever cultivated by humans, just 30 species provide around 95% of the world energy supply [4,5]. As food and nutritional security is still a distant dream, especially in many developing countries, finding a solution to assure food and nutritional security is a prime concern. If the current trend lasts, the number of people affected by hunger would surpass 840 million by 2030, against the target of achieving Zero Hunger by 2030 [3]. Economic disparity plays an important role in dietary habit.

**Figure 1:**
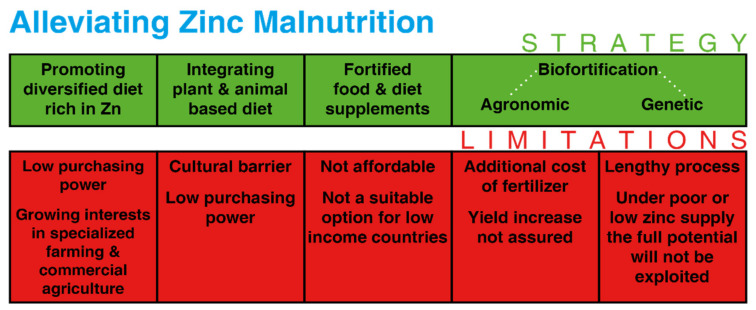
Different strategies for alleviating Zn deficiency.

Low-income countries largely trust staple foods while relying less on fruits, vegetables, and foods from animal sources compared to high-income countries. Micronutrient malnutrition, due to a lack of sufficient micronutrients in the diet results in serious but often invisible health consequences, referred to as hidden hunger. In the world, over 2 billion people are affected by micronutrient deficiency [6,7]. This micronutrient malnutrition can be attributed to consumption of a diet having low micronutrient content and low dietary diversity. Hidden hunger can be addressed through a nutrition-specific and nutrition-sensitive approaches [8]. The nutrition-specific approach or direct approach involves dietary interventions, i.e., consuming a more diversified diet, micronutrient supplements, and fortification. The nutrient-sensitive approach, on the other hand, involves the biofortification approach (Figure 1) [9]. Biofortification refers to the process of increasing the concentration and/or bioavailability of nutrients in the edible part of plants. Enhancing micronutrient concentration in the staples can be a sustainable and cost-effective intervention in improving the micronutrient uptake by people [10,11,12,13]. The most common source of food among people, especially in low incoming countries are cereal-based diets [14,15]; therefore, enriching them with micronutrients can improve micronutrient consumption. Staple food grains like wheat, rice, maize, etc. are consumed by a large number of people across the globe, when they are biofortified with Zn it could have a great impact in reducing hidden hunger.

Amongst different micronutrients, Zn inadequacy is common in both plants and animals [11,16]. Zn deficiency is considered an important risk factor for human health causing death [11,17]. Around 60–70% of the population in Asia and Sub-Saharan Africa could be vulnerable to low Zn uptake [18]. A third of the world’s population is at the risk of Zn deficiency, which is more predominant among children under the age of five, as they require more Zn to meet growth and developmental needs [15,19]. Zn deficiency is associated with several problems in human health, e.g., poor physical growth, weakened immunity system, DNA damage, cancer, increased risk of infections, poor birth outcomes in pregnant women, etc. [11,15,20,21,22]. The epidermal, nervous, gastrointestinal, skeletal, immune, and reproductive systems are the organs most affected clinically by Zn deficiency [23].

From the discussion above, it is very clear that a healthy diet can not only improve human health and maximize human resource potential. It can also reduce the costs related to health. We need suitable intervention like biofortification to make nutritionally superior food available to every person in the world at an affordable cost. In this direction, biofortification of staple food grains like rice, wheat, and maize with zinc and their inclusion in the human diet can help in alleviating zinc malnutrition.

## 2. Role of Zn in Human Health

Zn is a very crucial nutrient for maintaining optimal human health. The essentiality of Zn for human was established in the year 1961 [23]. Around 2800–3000 proteins in the human body contain Zn prosthetic groups [15,18,24]. Zn is also required for the function of over 300 enzymes [25]. It is also interesting that Zn is involved in all six classes of enzyme, i.e., hydrolases, lyases, ligases, isomerases, oxidoreductases, and transferases [26]. Zn plays a vital role in the overall physical growth, development, immune function, reproductive health, and neurobehavioral activity. Considering the diverse and very significant roles of Zn in human health, it can be safely assumed that Zn nutrition is of utmost importance for human health. As Zn performs multifaceted roles in the human body, various physiological signs are found in response to Zn deficiency. The response to Zn deficiency may vary depending on the extent/severity of the deficiency. The negative impact and clinical effect of Zn deficiency may vary with age; diarrhoea is the most common symptom in early infancy [23]. Skin related problems, recurrent infections, and dwarfing are common among toddlers or school-aged children [18,23,27]. The manifestation of Zn deficiency among adults includes recurrent infections, hypogeusia, chronic non-healing leg ulcers, and adverse pregnancy outcomes [18,23].

Though the mechanism of Zn deficiency-induced impairment of growth and development is not clearly understood, it is one of the most studied effects of Zn deficiency. The effect of Zn deficiency is more significant especially in the time of rapid growth like infancy, puberty, and pregnancy [23]. Intake of a supplementary dose of Zn was found to minimize the risk of malaria [28], pneumonia [29], and diarrhoea [23,29].

Considering the importance of Zn in human health (Table 1), there is a growing interest in improving Zn nutrition. Strategies are being made to improve Zn intake among humans to improve the bioavailability of the Zn [30,31,32]. Bioavailability refers to the fraction of Zn intake that can be absorbed in the human gut [33]. Thus, not only Zn intake but also its bioavailability should be given equal importance for improving Zn nutrition [7,11]. The bioavailability of Zn can be improved by reducing the anti-nutrition factor-like phytate in the grain [15]. The people in developing countries where the diet is dominated by cereals are subjected to micronutrient malnutrition disproportionately, since cereals are not only a poor source of micronutrients but also the high content of phytate in cereals reduces the bioavailability of Zn further [33,34].

## 3. Role of Zn in Crop

Zn is an essential plant nutrient and known as a micronutrient because of its low requirement. Zn remains in plants in either free ionic form or as a complex with many low molecular weight compounds [48]. Though the requirement of Zn in plants is low, it plays an important role in overall plant growth and development [33]. Zn is essential for different biochemical processes including auxin metabolism [48], chlorophyll synthesis [33], and activation of different enzymes. Zn is also involved in carbohydrate [48], lipid, and nucleic acid metabolism [7].

Zn is a constituent of many important enzymes of great significance such as carbonic anhydrase, alcohol dehydrogenase, and superoxide dismutase [33,49]. Carbonic anhydrase facilitates photosynthetic carbon dioxide fixation [48]. This may be one of the reasons why photosynthesis is inhibited under acute Zn deficiency. Alcohol dehydrogenase performs a vital job in flooding tolerance of crops [50,51]. Superoxide dismutase helps in the detoxification of superoxide radicals and protects the lipids and proteins of the membrane against oxidation [52,53]. Lack of sufficient Zn in the plant may damage the membrane integrity and increases membrane leakiness.

Zn is also an integral part of the Zn finger family of transcription factors that controls cell proliferation and differentiation [33]. Zn plays a vital role in chloroplast function and development, where Zn dependent SPP peptidase activity and Photosystem II repairing are very crucial [33,54]. Zn deficiency causes a significant decline in the chlorophyll content and abnormality in chloroplast structure, thus negatively affecting photosynthesis in plants [48].

The possible role of Zn in water uptake and transport in plants and a short-term tolerance to heat and stress tolerance has been reported [55,56,57,58,59]. The function of Zn finger proteins in stress tolerance was reported by many researchers [60,61,62,63]. The role of Zn in plant defence against pathogens and herbivores have also been highlighted [64].

## 4. Biofortification for Grain Zn Enrichment: The Concept

Zn deficiency in crops limits crop yield and grain nutritional quality [11,65]. Cereal crops, which play a significant role in meeting the daily calorific need in developing countries, are usually low in Zn concentration [9,15]. The Zn concentration in cereals is found to be even lower when the crop is grown in soil with low Zn content [11]. The regions having low soil Zn concentration shows a prevalence of Zn deficiency among humans suggesting a strong interrelationship among soil–plant–human health [11]. The prevalence of high Zn deficiency in soils might be due to low addition of organic matter to soils, intensive agriculture that removes a huge amount of nutrient from the soil including micronutrients, and lower use of micronutrient fertilizers. Biofortification attempts to improve this soil–crop–human interrelationship in a way that can ultimately help to alleviate Zn deficiency in humans [9].

The biofortification approach aims at enriching the grains with minerals like iron, Zn, selenium, iodine, etc., so that, their intake can be improved among people consuming those grains [6,9,14]. As micronutrient malnutrition or hidden hunger is more prevalent in low-income countries where consumers have low purchasing power, consequently they can hardly afford micronutrient supplements or a relatively healthier diet rich in micronutrients. Under such conditions, improving the nutrient status of the commonly consumed staple food grains gives the most sustainable option for alleviating micronutrient malnutrition [11,34,66]. An effective biofortification strategy should ensure that grain yield be improved or at least maintained, increase the grain Zn concentration for significant human health benefits, and the grain performance must be stable across environments [16,67]. As Zn deficiency is often associated with a cereal-based diet and because people in developing countries—especially in rural areas—are highly dependent on cereal-based diets due to reasons such as, poor purchasing power, cultural preference, and high food price, biofortification of cereal grains with Zn can be a sustainable solution to increase Zn intake [14,15].

Understanding the physiological basis of micronutrient accumulation in the grains is of utmost importance for successful biofortification. Micronutrient concentration in the edible portion of the crop is decided by nutrient availability to plants and the process of absorption, translocation, and redistribution of micronutrients in the plant, which are being regulated by homeostatic mechanisms that allow accumulation of micronutrients in an adequate amount, yet at a non-toxic level [9,16]. The absorption of nutrients by the plant depends on soil factors, e.g., the physicochemical and biological properties of soil, agronomic factors, or management factors, that decide micronutrient availability at rhizosphere and plant factors such as root morphology, root cell activities leading to increased micronutrient solubility and mobility [10,18,48]. All these factors are in a close and complex interaction with each other that decides micronutrient absorption by plants. The agronomic practices like organic matter addition, micronutrient fertilizer application, maintaining optimum soil moisture for facilitating Zn diffusion to plant roots can help in increasing micronutrient absorption by plant roots [18].

The crop also plays a very important role in the absorption of micronutrients. For enhancing Zn uptake, the micronutrient level in the root–soil interface should be increased. Changing root morphology for increasing the absorptive surface area can enhance Zn uptake. Efflux of H^+^ from root cells and the release of metal complexing compounds and reductants can also be very useful root characteristics for increasing Zn uptake [16]. Understanding these traits can help in developing new Zn-efficient genotypes. After uptake, the micronutrient must be efficiently translocated and accumulated in the edible parts of the plant [9]. The micronutrients can be translocated either directly from the roots to the grains or they can be re-translocated from the vegetative tissues after the reproductive period [9,11]. The process of translocation and re-translocation, concerning different genotypes and environmental conditions, must be studied. The accumulated micronutrients should be bioavailable to give a favourable health outcome for the people consuming the biofortified food grains. Two approaches of biofortification, agronomic biofortification and genetic biofortification are discussed below:

### 4.1. Agronomic Biofortification

When the soil is inherently deficient in Zn content and/or the solubility of Zn in soils is low, then the plant cannot absorb sufficient Zn to meet its physiological or metabolic need. In fact, nearly 50% of cereal growing areas in the world have been found deficient in Zn [11]. This leads to low Zn concentration in the grains of crops grown in those soils. Under such a scenario, improving the availability of Zn to the crop can improve grain Zn concentration [4,6,7,10]. This simple approach of crop fertilization with Zn-based fertilizers, to improve grain Zn concentration, is termed agronomic biofortification. Application of Zn-based fertilizers to the crop shows different responses depending on the method of application (e.g., soil/foliar/seed priming or any combination of different methods), source of Zn applied time of Zn application, and also the genetic makeup of the crop and the environment in which the crop is grown [7,15,17,18]. Considering these large numbers of factors, which can potentially affect the efficacy of agronomic biofortification in improving grain Zn concentration, the source, time, method of application, and rate of application must be optimized to get the best possible result [7,12,18].

Agronomic biofortification is cheap and provides the dual advantage of yield enhancement and improvement of grain Zn concentration. Though genetic biofortification also shows a lot of promise, developing a variety takes a lot of time and effort [11]. Moreover, the achievement of genetic biofortification may be jeopardized in the absence of sufficient Zn in the soil [11].

#### 4.1.1. Effect of Different Methods of Zn Application on Grain Zn Enrichment

Zn is provided to the crops by soil application, foliar application, seed application (priming), or by a combination of these methods [68,69,70,71,72]. Different responses are found with different application methods. Each application method has certain advantages and limitations (Table 2). Soil application is usually the most commonly used method of soil application. The efficacy of soil-applied fertilizer largely depends on the soil environment (pH, moisture content, presence of antagonist nutrients, etc.) and ability of plants to successfully absorb the nutrients whereas in the foliar application, the uptake and translocation of nutrients to the grain is largely dependent on the crop. Different crops show different responses to the Zn application methods. For example, among rice, wheat, and maize, the response of wheat (in terms of increase in grain Zn concentration) to foliar Zn application was highest, followed by rice and maize [15].

#### 4.1.2. Soil Application

The efficiency of soil-applied Zn fertilizer depends on soil pH. The availability of Zn is relatively higher at acidic soil pH. The solubility of soil decreases hundred-fold with each unit increase in pH [73]. Liming of acidic soils has been found to reduce the availability of Zn [18]. The Zn deficiency in calcareous soil can be attributed to a CaCO_3_ induced rise in pH, direct sorption of Zn to the precipitated CaCO_3_, and formation of insoluble calcium Znate [22]. Alkaline soil, covering about 30% of the global farmland, shows low Zn availability to plants [15,74]. Other than pH, the other factors influencing Zn fixation are complexation with organic matter, occlusion in minerals, diffusion into micropores and interparticle space, solid-phase diffusion, and co-precipitation with other metals [18,75,76].

As Zn reaches the plant root predominantly through diffusion [77], therefore low soil moisture and organic matter limits the process and thus plant Zn availability is reduced [11,15,78,79]. As limiting moisture condition and low organic matter is a common condition observed in agricultural fields, consequently such conditions are expected to reduce Zn uptake by plant roots [74,80]. Soil moisture plays an important role in soils with low Zn availability. The soil moisture acts as a medium for Zn transport from soil to root in the diffusion process. Hence, the negative impacts of Zn deficiency are more fatal under rainfed conditions than under irrigated condition [11]. The Zn availability to crops from soil-applied Zn may also be affected by its interaction with other nutrients. Positive Zn–nitrogen interaction has been reported by many researchers [18,81,82]. The negative Zn–phosphorus interaction is one of the most widely studied nutrient interactions [18,83,84]. Excess phosphorus in the soil reduces the Zn availability to a plant. A decrease in uptake of Zn due to a high application dose of phosphorus might be due to reduced Zn concentration in soil solution and reduction in VAM infection resulting decrease in Zn uptake [18]. Zn has also been found to interact negatively with Fe, Mn, and Cu [18]. These interactions are to be considered in understanding the availability of Zn to plant. Soil moisture content has also a significant effect on zin availability. Soil moisture content affects Zn availability by modifying redox potential, pH, and dissolved organic anions [85]. In addition to the chemical characteristics of soil, the biological characters of soil also play a vital role in deciding Zn availability to plants [18,85]. PGPRs refers to a group of bacteria, which have many positive impacts on plant growth [86]. They have the capacity of improving mobility and uptake of nutrients [18,87,88] and have been found to be suitable in improving Zn availability to plants. The presence of *Arbuscular mycorrhiza* in soil also plays an important role in mobilizing Zn to plants [89]. The effectiveness of soil-applied Zn, like any method of Zn application, also depends on the genetic characters of the plant. The source of zinc and dose of soil zinc application has also been found to affect the outcome in terms of yield and grain quality [7,18]. The optimum dose of soil zinc application may vary depending on the crop and soil zinc status. While recommending a zinc dose, care should be taken to avoid zinc toxicity to plants.

#### 4.1.3. Foliar Application

The efficiency of foliar Zn application greatly depends on the type of fertilizer, crop characteristics, especially, leaf characteristics and genetic potential of the crop [7]. The foliar application gives multiple advantages, such as comparatively low fertilizer requirement, no Zn fixation, and no influence of antagonist nutrient in the uptake of Zn [18]. Foliar applied Zn is phloem mobile [15] and can be transferred to developing grains. In a crop like wheat, foliar application of Zn has been found to be superior in increasing grain Zn concentration as compared to soil application [4,67,68]. The effectiveness of foliar application on increasing grain Zn concentration varies significantly with the time of application [11].

**Figure 2:**
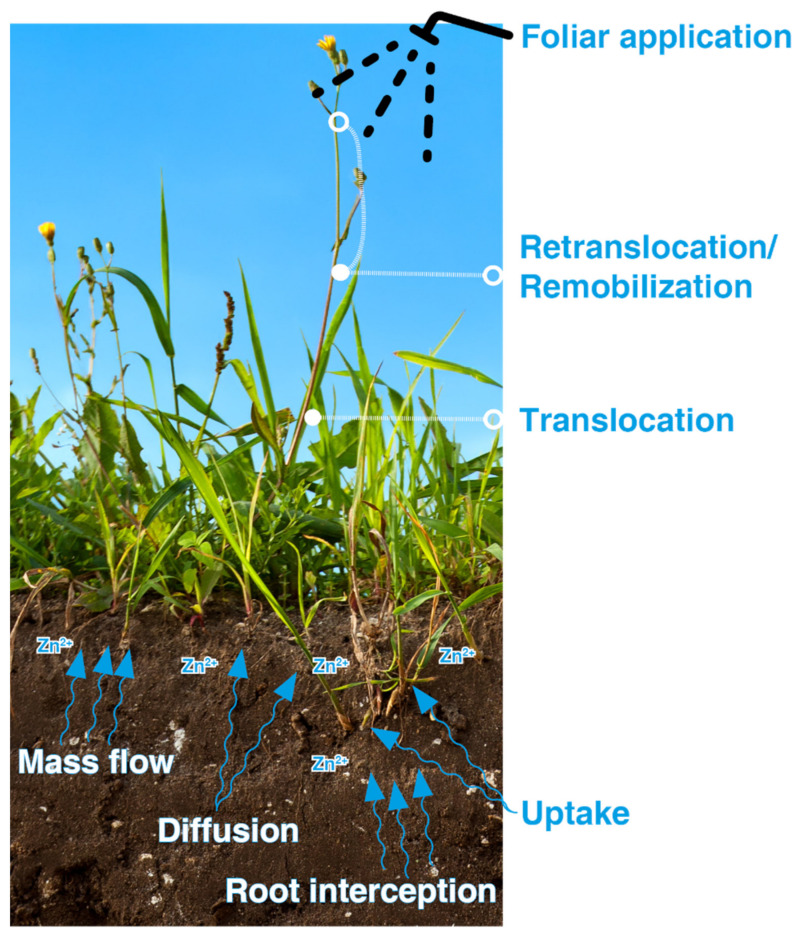
Uptake, translocation, and remobilization of Zn in plants.

A poor correlation between DTPA-Zn concentration of soil and grain Zn concentration has been reported [67]. This lack of correlation may be due to the unfavourable soil conditions that limit their mobilization of nutrients to plant roots and hence their subsequent absorption. As the foliar application does not interact with soil, they are not subjected to any form of fixation. Under unfavourable field conditions, grain Zn concentration is greatly reliant on the remobilization of this trace element from vegetative tissues (Figure 2) [7,67], hence maintaining a higher concentration of Zn in the vegetative tissue may improve the grain Zn concentration [82,90]. Unfavourable field conditions are very normal; therefore, foliar application of Zn can be a very useful option to increase grain Zn concentration.

The time of foliar application is very critical for the effectiveness of foliar-applied Zn [91,92]. Higher grain Zn enrichment is achieved when Zn is applied at a later growth stage [91]. Application of Zn at the heading and early milk stage was found to be more effective than foliar application of Zn at the booting and stem elongation stage [92]. The higher effectiveness of Zn applied at the milking stage through the foliar application method might be because of active photo-assimilation allocation to the reproductive sink that caused the mobilization of micronutrients to the sink organs. Stronger phloem mobility of Zn for foliar application is observed during the reproductive stages [92,93]. Foliar application of 0.5% (*w*/*v*) ZnSO_4_. 7H_2_O at the heading and milk stage of wheat resulted in a significant increase in grain Zn concentration across locations in seven different countries over 2 years of an experiment. The result suggested an average increase in Zn concentration by 83.5%, while soil Zn application showed an average increase of 12.3% over no Zn application [67]. Foliar spray of Zn has also been observed in increasing grain productivity under drought conditions [94] and such improvement in yield under drought condition might be due to improved defence mechanism against stress-induced oxidative cell damage [67,95].

#### 4.1.4. Seed Application

High seed Zn content has been found to improve seedling vigour and crop stand in the field [96]. When plants are grown in a nutrient deficient area then seeds produced will be deficient in nutrients and when such seeds are again resown in a nutrient deficient area then overall seedling vigour, growth, and yield of plants is reduced [96]. As Zn is transported to plant roots through diffusion, moisture deficiency affects the Zn availability to plant [18]. Thus, soil-applied Zn may not be equally effective in moisture deficient conditions and irrigated conditions. Under the rainfed condition where soil moisture content solely relies on rainfall, the result of Zn nutrition can be highly inconsistent. Seeds rich in Zn content could improve plant growth and yield under Zn deficient condition especially under rainfed condition [96].

Seed priming of wheat with Zn significantly increased grain Zn concentration by 12%, while the concentration of chickpea and maize improved by 29% and 19%, respectively [97]. Seed priming was also found to be cost-effective in all three crops, i.e., wheat, chickpea, and maize [97]. Unlike a foliar application, the effect of seed priming has been noted as less effective in improving grain Zn concentration [7]; however, they may play an important role especially under resource-poor conditions and stressed environments.

#### 4.1.5. Combination of Application Methods

Different combinations of application methods (soil + foliar and seed + foliar) have also been studied to show their effectiveness in improving grain zinc concentration [68,70,98]. Combination of soil and foliar application of Zn has been found to increase the grain Zn concentration compared to soil or foliar application alone in few experimental locations; while in most of the experimental locations, the combined application showed an at par result with the foliar application alone [67]. Improvement in grain Zn concentration of durum wheat by the combination of soil and foliar application was higher as compared to soil application alone [4].

#### 4.1.6. Other Agronomic Practices to Improve Zn Uptake

Nutrient uptake from different application methods may vary due to environmental (soil and atmospheric) and crop characteristics (morphological, physiological, genetic). Agronomic management plays an important role in altering the crop environment, thus agronomic management practices other than Zn fertilization are also expected to affect Zn uptake.

The moisture status of soil may vary depending on the irrigation practices followed. Moisture deficiency in crop field reduces the diffusion of Zn, limiting their absorption by the plant. Zn deficiency has been observed in rice under contrasting environments, i.e., flooded conditions as well as aerobic conditions [18]. In aerobic rice, the soil has more NO^3−^ ion resulting in more release of OH^-^ from rice roots, which ultimately precipitates Zn thus reducing its’ level [99].

In addition to Zn fertilization, the status of other nutrients in the soil and their application also affects Zn uptake. Nutrient interaction may alter the availability of nutrients either positively or negatively. A sufficient level of nitrogen supply is very critical for increasing the grain Zn concentration of wheat. Nitrogen application improves uptake as well as remobilization of Zn in wheat [100]. The role of nitrogen in improving Zn biofortification of maize was reported [101]. Unlike nitrogen, phosphorus plays an antagonistic role with Zn [83,102]. Zn has also been found to interact with other micronutrients like iron [103,104,105], boron [106,107], and copper [108]. The effect of different interactions should be studied to develop a proper Zn application strategy.

#### 4.1.7. Additional Benefits of Zn Fertilization

In addition to enhancing grain Zn concentration and sometimes yield, agronomic biofortification gives some additional benefits. The soil application of Zn under a Zn deficient condition has been found to reduce the uptake and accumulation of phosphorus. This Zn–phosphorus antagonism is one of the most discussed nutrient antagonisms. The reduced phosphorus uptake and accumulation may reduce the phytate content in grains [11]. Under Zn deficiency, the uptake and shoot accumulation of phosphorus increases, which results in a corresponding increase of phosphorus in grain due to high phloem mobility of phosphorus [11,109]. Most of the inorganic phosphorus in grains is transformed into phytic acid. As phytate is considered as an antinutritional factor and reduces the bioavailability of Zn [110,111], low phytic acid in grains will improve Zn bioavailability. The phytate–Zn molar ratio is used as an indicator for estimating Zn bioavailability [112].

In addition to human health benefits, grains enriched with Zn can give additional agronomic benefits. Seeds with low Zn concentration show poor tolerance to environmental stresses [113]. When seeds with low Zn concentration are grown on Zn deficient soil, it results in poor crop establishment and seedling vigour [96]. Maintaining a sufficient level of seed Zn also provides defence against soil-borne pathogens [11]. As Zn rich seeds improve seedling vigour and crop establishment the seeding rate may be decreased [11,114].

### 4.2. Genetic Biofortification

The second approach for enhancing grain Zn concentration in crops is genetic biofortification. Genetic biofortification follows the breeding approach to increase the concentration and bioavailability of grain Zn. Genetic biofortification can serve as a cost-effective strategy to alleviate Zn deficiency. A superior genotype, once developed, can be used for many years without any additional recurring cost.

#### 4.2.1. Strategies for Genetic Biofortification

Plant breeding and/or transgenic approaches provide a hopeful and long-term strategy to overcome micronutrient malnutrition by developing genotypes with a high level of Zn in the edible plant parts [16,34,67]. Though the cost of developing a genotype is costly and time-consuming, it gives a long term benefit as it does not involve any recurring cost. Breeding for high grain Zn concentration is possible as sufficient genetic variation is found in the germplasms of major cereal crops [115].

The overall steps involved in breeding include the following minimum steps: finding suitable genetic variation and selection of parents, long term crossing and backcrossing, stabilization of target traits across multiple environments/climatic conditions, and adaptation of the biofortified genotypes to the regional agronomic management practices [11]. The breeding criteria for micronutrient enriched food crops as outlined by Welch and Graham [16] are: crop productivity must be maintained or increased, achieve a micronutrient level that can have a significant impact on human health, stability of micronutrient enrichment traits across the various edaphic environment and climatic zones, the bioavailability of micronutrients in humans should be sufficient to improve micronutrient levels of people “preparing and eating them in traditional ways within normal household environments”, and consumer acceptance must be tested. In the past century, a significant increase in grain yield was observed owing to a breeding strategy especially attempting to increase crop productivity and improved agronomic practices. Such an increase in yield was very marked during the green revolution period. With the rise in grain yield, a considerable decrease in the grain Zn was observed due to the “dilution effect” [15,116,117].

A superior genotype for Zn biofortification needs to have the following characteristics: high Zn acquisition efficiency, readily translocate Zn to grain/edible part of plant, efficient remobilization of Zn from vegetative tissues to grain or edible part of the plant, and availability of Zn in the plant in a bioavailable form that can be utilized by the person consuming it [14,118]. Several genes are involved in controlling those characteristics. As the performance of any type is affected by the environment the performance stability of the genotypes must be evaluated at multiple locations over a reasonable period to confirm their efficiency. While breeding biofortified varieties, care must be taken not to compromise the end-use characteristics so that it can be quickly adopted by consumers as well as producers [4]. As consumer preference varies over locations care must be taken to develop a variety that appeals to consumer taste or sensory preferences.

Existing genetic variability, trait heritability, gene action, the association among traits, available screening techniques, and diagnostic tools are commonly used criteria to estimate the potential genetic gains [4]. A large genetic variation for grain Zn concentration exists among modern wheat genotypes and their wild relatives [11,67]. This genetic variation can be beneficially exploited under different breeding programs. Combining high grain Zn concentrations with a high yield under different environmental conditions is very important for a successful genetic biofortification strategy.

In addition to grain Zn concentration, the bioavailability of the Zn should be given importance in the breeding programs. Only 25% of the Zn in the staple food grains are thought to be bioavailable [14]. The bioavailability of micronutrients is often limited by antinutrient factors like phytic acid. Though genotypes can be bred for low antinutritional factor, care should be taken as many antinutritional factors play role in plant metabolism and provide resistance against biotic and abiotic stress [14,119]. Moreover, antinutrients like polyphenols and phytate in human diets provide multiple health benefits by acting as an anti-carcinogen and antidiabetic [120,121,122]. Phytic acid in seeds also plays an important role in seed germination and good seed vigour [11,123,124]. Hence, a proper balance should be found to get the benefits of antinutritional factors while maintaining sufficient bioavailable Zn level in the grain. The Zn that will actually be bioavailable after harvesting, processing, and cooking needs to be evaluated to get a clearer picture of the actual benefit that can be harnessed through biofortification.

A transgenic approach can be followed to develop crop varieties with a high Zn content. Evidence of ZIP family iron and Zn transporter proteins in improving grain micronutrient concentration is available [11,125,126]. These transporter proteins are involved in the uptake and transport of cationic micronutrients. The expression of a Zn transporter gene from *Arabidopsis thaliana* in barley roots increased the Zn concentration in grain [127].

#### 4.2.2. Limitations and Constraints of Genetic Biofortification

In the agronomic biofortification section, we have briefly discussed how various soil characteristics affect a plant’s availability of Zn. Under such a condition, the varieties developed to accumulate more Zn in the edible parts may not be able to show its full potential. To achieve a grain Zn concentration in the edible portions of the plant that can bring a measurable biological impact, the plant must be grown in a soil environment with sufficient plant-available Zn [11]. As the majority of the world soils under cereal cultivation have adverse chemical properties Zn nutrition to crops grown under such condition is impaired [11,80,128,129].

Moreover, crop improvement to develop Zn rich variety is a fairly lengthy process and requires a lot of effort in germplasm selection/screening, crossing, and performance assessment in the multilocation trial. However, as Zn concentration is not subjected to genetic erosion, little maintenance breeding is needed after the incorporation of the desired gene into the gene pool [4].

## 5. Combining Agronomic and Genetic Biofortification

**Figure 3:**
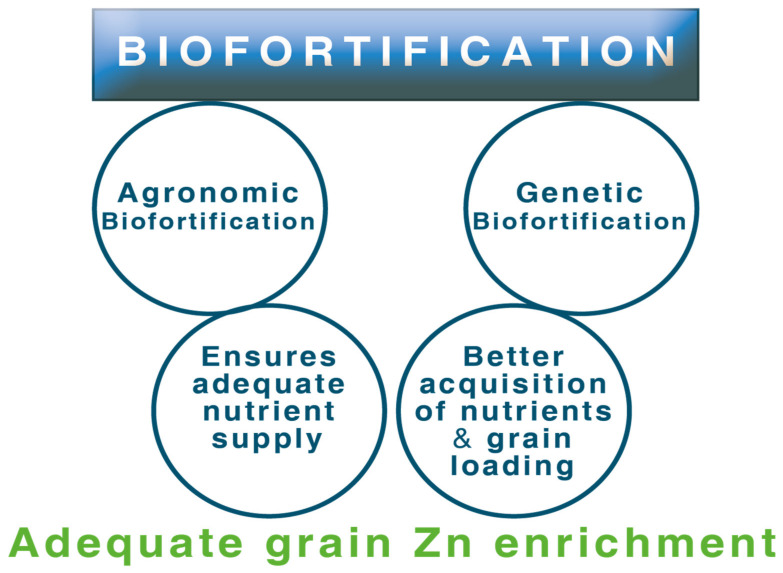
Complimentary effect of agronomic and genetic biofortification.

The success of genetic biofortification may be jeopardized if sufficient Zn is not available in soil [11]. The capacity of plants to absorb sufficient Zn from the soil will not be enough unless there is sufficient Zn in the soil available to be absorbed. Hence, a suitable genotype capable of absorption and translocation of Zn towards the grain efficiently when supplied with sufficient plant-available Zn is expected to give the best possible result (Figure 3). As genetic biofortification takes a comparatively longer time, agronomic biofortification may serve as a complementary approach to achieve high grain Zn to some extent. Selection of suitable variety when combined with the right application method and right fertilizer application method could improve micronutrient concentration in different crops [92].

## 6. Economic Points of View for Zn Biofortification

The unavailability and unaffordability of a healthy diet are largely responsible for the prevalence of malnutrition across the globe. As per an estimate, over 3 billion people worldwide are far from easy access to a healthy diet. Thus, both accessibility and affordability of a healthy diet must be ensured. By 2030, diet-related health cost linked to death and non-communicable diseases are expected to exceed USD 1.3 trillion while, the diet-related social cost of GHG (greenhouse gas) emission is projected to be greater than USD 1.7 trillion. Though these costs are often ignored, a dietary shift to a healthy diet can reduce the cost related to health and climate change. In fact, adoption of a healthy diet can reduce the direct and indirect health costs up to 97% and a 41–74% reduction in social cost in GHG emission by 2030 [3].

As the application of Zn may not necessarily increase the yield, farmers may be sceptical to apply Zn as it incurs an additional cost. However, the health benefits obtained from Zn application are usually overlooked in such conditions. Zn and other micronutrient deficiency cause huge economic losses in developing countries and have a huge impact on gross domestic product (GDP) and costs related to health care [15,130]. Micronutrient deficiency is responsible for economic cost at the individual, community, and national levels [130]. Hence, an appropriate policy to encourage and incentivize farmers for producing Zn enriched grains is required.

Wang et al. [131] calculated the cost-effectiveness of agronomic biofortification by using the “disability-adjusted life year” to calculate the health issues. They found that agronomic biofortification of wheat with Zn could improve the dietary intake of Zn among infants and children below five-years of age, consequently reducing Zn deficiency-related health burdens by up to 56.6% in the study region. They also showed that US $226 to US $594 is required to save one “disability-adjusted life year” when the foliar spray of Zn is done alone, while, with foliar application of Zn fertilizer is combined with pesticide spray, the labour cost drops, and only US $41 to US $108 is required to save one “disability-adjusted life year”.

The suboptimal utilization of the human resource potential is expected to reduce the work productivity and thus will have an undesirable impact on economic output at all levels, starting from the individual to the national level. Intervention is required to reduce micronutrient malnutrition so that human resource potential can be fully utilized to their potential and cost of health can be reduced. Biofortification may be seen as an investment in human health, which will also reduce the cost of health. Unless we evaluate this health benefit of biofortified grains and see grain yield as the only criteria for evaluating the economic output of crop production; then the benefits of biofortification can be hardly realized. Social awareness on the importance of micronutrient nutrition, policies to promote micronutrient application in crops to realize the benefits of agronomic and/or genetic biofortification, and investment in the research and development on biofortification can help in sustainably alleviating micronutrient malnutrition.

## 7. Future Scopes

Though research has advanced in biofortification, some key areas need to be addressed or improved further. Some have been highlighted below:

- A comprehensive 4R (right place, right time, right source, and right dose) approach of Zn application can be developed for different crops at the regional level and the best combination can be found for achieving high grain Zn concentration.
- Physiological constraints of grain Zn accumulation must be identified for different crops under different conditions and agronomic and genetic approaches for ameliorating these constraints may be found to further improve the grain Zn density.
- Biofortification options must be studied under stressed environments and their effects must be evaluated under such conditions. As climate change is expected to bring more weather anomalies, a stress-proof biofortification approach must be developed.
- The bioavailability of Zn obtained through foliar application can be compared with other application methods. Agronomic management that improves grain Zn bioavailability should be studied.
- The environmental implications of continuous Zn application should be studied. Continuous application of Zn over a long period may cause Zn toxicity and therefore should be regularly monitored.
- The performance of Zn-efficient genotypes under different soil Zn availability should be evaluated. The beneficial effects of combining the agronomic and genetic biofortification approach should be explored.

## 8. Conclusions

From this comprehensive review, it can be concluded that the biofortification approach has outstanding potential for ameliorating the problem of micronutrient malnutrition. The cost-effectiveness of this approach makes it a suitable option for low-income countries. As biofortification improves the micronutrient concentration of the staple food grains that is predominantly consumed by people it does not involve any dietary change and can be adapted by people quickly. Agronomic biofortification not only improves the grain Zn concentration providing health benefits, but it can also help in reducing the extent of Zn deficiency especially in regions where intensive cropping is practiced and micronutrient application is overlooked. With the advent of genetic engineering and molecular tools, the breeding approach of biofortification can also be fastened and developing a superior Zn-efficient and Zn-rich varieties will be comparatively easier to find. Policy initiative and government support will also help in further research and dissemination of biofortification technologies and practices.

## Figures and Tables

**Table 1:** Role of Zn in different organ systems.

Organ Systems	Role of Zn	References
Cardiovascular	A cardioprotective role, reduces risk of heart failure, and an important role in cardiovascular health	[35,36]
Integumentary system	Skin health, wound healing, protection against UV radiation, and acts as antioxidant	[37,38,39,40]
Reproductive system	An important role in formation and maturation of spermatozoa, for ovulation and fertilization; functioning of the male and female reproductive system	[41,42,43]
Nervous system	The modulator of neuronal excitability, an important role in neuronal metabolism, and the modulator of synaptic activity and neuronal plasticity	[44,45]
Respiratory system	Reduces the incidence of acute lower respiratory infection; low Zn may increase the risk of pneumonia in elderly	[46,47]
Endocrine system	Thyroid hormone metabolism, structure, and activity of insulin.	[43]

**Table 2:** Different Zn application methods, their advantages and limitations.

Application Methods	Advantages	Limitations
Soil Application	Minimizes soil Zn deficiency The residual effect may benefit subsequent crops	High fertilizer requirement Availability to plant may decrease due to adverse soil properties
Foliar application	Lower fertilizer requirement Not affected by adverse soil characteristics	Crop requirement in the early seedling stage is not met Very high dose of nutrient cannot be applied using the foliar method
Seed priming	Lower fertilizer requirement Suitable for stressed environments	A higher amount of nutrient cannot be applied using this method as a high concentration of priming solution may negatively affect germination

## Data Availability

Most of the recorded data are available in the Tables and Figures of the manuscript.
